# Prevalence and Correlates of HIV and Hepatitis C Virus Infections and Risk Behaviors among Malaysian Fishermen

**DOI:** 10.1371/journal.pone.0118422

**Published:** 2015-08-05

**Authors:** Martin K. K. Choo, Nabila El-Bassel, Philippe C. G. Adam, Louisa Gilbert, Elwin Wu, Brooke S. West, Alexander R. Bazazi, John B. F. De Wit, Rusli Ismail, Adeeba Kamarulzaman

**Affiliations:** 1 Centre of Excellence for Research in AIDS (CERiA), Faculty of Medicine, University of Malaya, Kuala Lumpur, Malaysia; 2 Social Intervention Group, Columbia University School of Social Work, New York, New York, United States of America; 3 Centre for Social Research in Health, Faculty of Arts and Social Sciences, UNSW Australia, Sydney, Australia; 4 Department of Epidemiology of Microbial Diseases, Yale School of Public Health, New Haven, Connecticut, United States of America; University College London, UNITED KINGDOM

## Abstract

Fishermen in Southeast Asia have been found to be highly vulnerable to HIV, with research evidence highlighting the role of sexual risk behaviors. This study aims to estimate the rate of HIV as well as hepatitis C virus (HCV) infections among Malaysian fishermen, and the risky sexual and injection drug use behaviors that may contribute to these infections. The study also includes an assessment of socio-demographic, occupational and behavioral correlates of testing positive for HIV or HCV, and socio-demographic and occupational correlates of risk behaviors. The study had a cross-sectional design and recruited 406 fishermen through respondent-driven sampling (RDS). Participants self-completed a questionnaire and provided biological specimens for HIV and HCV testing. We conducted and compared results of analyses of both unweighted data and data weighted with the Respondent-Driven Sampling Analysis Tool (RDSAT). Of the participating fishermen, 12.4% were HIV positive and 48.6% had HCV infection. Contrary to expectations and findings from previous research, most fishermen (77.1%) were not sexually active. More than a third had a history of injection drug use, which often occurred during fishing trips on commercial vessels and during longer stays at sea. Of the fishermen who injected drugs, 42.5% reported unsafe injection practices in the past month. Reporting a history of injection drug use increased the odds of testing HIV positive by more than 6 times (AOR = 6.22, 95% CIs [2.74, 14.13]). Most fishermen who injected drugs tested positive for HCV. HCV infection was significantly associated with injection drug use, being older than 25 years, working on a commercial vessel and spending four or more days at sea per fishing trip. There is an urgent need to strengthen current harm reduction and drug treatment programs for Malaysian fishermen who inject drugs, especially among fishermen who work on commercial vessels and engage in deep-sea fishing.

## Introduction

Fisheries in Southeast Asia employ an estimated thirty million people, supply one quarter of global fish production annually, and are key contributors to rural livelihood, export revenue, and food security.[1–3] A review of available data from Southeast Asian countries found HIV prevalence rates among fishing communities to be between 4.6 and 14 times higher than in the general population.[4] Findings that Southeast Asian fishermen are highly vulnerable to HIV have raised serious implications for public health, society and the economy.[4,5] There is however a paucity of data on the dynamics of the HIV epidemic among fishermen in this region to inform prevention efforts.[4,6,7]

Previous prevalence estimates suggest that as many as 15.5% of fishermen in Thailand may be infected with HIV, and HIV prevalence among fishermen in Cambodia was estimated at a comparable 16.1%.[8,9] Risky sexual behavior has been found to be the primary route of HIV transmission among fishermen,[10] including in Thailand and Cambodia, with scant evidence of injection drug use playing a role.[4,8,9] In Malaysia, fishermen are estimated to account for 3.5% of the 107,714 cumulative HIV diagnoses reported between 1986 and 2010. As fishermen represent only 1.3% of the Malaysian workforce, the HIV burden in this population group is disproportionately high.[11,12] To date no empirical studies have directly examined the burden of HIV among fishermen in Malaysia, nor the risk behaviors that may drive these HIV infections. Field reports of HIV prevention programs in Malaysia note that fishermen make up a large proportion of clients of needle and syringe exchange programs (NSEPs) on the east coast of Peninsular Malaysia, raising the possibility that the high burden of HIV among fishermen in Malaysia may result from injection drug use.

This paper reports on a respondent driven sampling (RDS) study of HIV and hepatitis C virus (HCV) infection, as well as sexual risk behaviors and risk behaviors related to injection drug use among a sample of fishermen in Kuantan, Malaysia. RDS was undertaken because fishermen, including those who work in small-scale fisheries, exhibit characteristics of hard-to-reach and hidden populations previously amenable to RDS methodology.[13,14] We computed population estimates using RDS data and compared unweighted data with RDS weighted data in statistical analyses. We also investigated socio-demographic, occupational and behavioral correlates of HIV and HCV infection, including sexual behaviors and injection drug use. Guided by anecdotal evidence, we hypothesized that, unlike in other Southeast Asian countries where HIV transmission appears to be mostly driven by sexual practices,[8,9] injection drug use is a main driver of HIV as well as HCV infections among fishermen in Malaysia. In addition to high rates of HCV infection among people who inject drugs (PWID), previous research has also found that rates of HIV may be high among PWID in many countries.[15,16] It is estimated that in Malaysia HIV prevalence in PWID is 10.3%.[16] We also examine why HIV and HCV risk resulting from injection drug use may be high among Malaysian fishermen and discuss implications for harm reduction and drug treatment programs targeting fishermen and their communities.

## Methods

### Ethics Statement

Participants provided written informed consent prior to study enrolment. The study was approved by the University of Malaya Ethics Committee and the Columbia University Institutional Review Board.

### Study Site

We conducted this study between July and December 2011, among fishermen in Kuantan, a port town on the east coast of Peninsular Malaysia approximately 300km from Kuala Lumpur. Kuantan was among the first towns in Malaysia to adopt an NSEP in 2006.

### Population

Malaysian government statistics identified 3,720 registered fishermen in Kuantan in 2009,[12] but no adequate sampling frame was available that included unregistered, typically small-scale, artisanal fishermen. This is common globally, given the lack of a universally accepted definition and consensus regarding what constitutes small-scale fisheries,[2] resulting in a an underestimation of the number of small-scale fishermen. Worldwide, the number of small-scale artisanal fishermen is thought to substantially outnumber large-scale commercial fishermen and small-scale fishermen may contribute more than half of the global fish catch.[17]

### Sampling and Data Collection Procedures

We used RDS, a chain referral sampling methodology, for participant recruitment.[13,14] Recruitment coupons with unique identifiers were used to track peer referral and link recruitment data with survey and serological data.

Three sampling locations were selected: two government-owned commercial fishery wharfs in Kuantan (designated wharfs A and B), and a fishing village within a 100km radius of Kuantan. Wharf A was used by fishermen working on commercial vessels of various sizes, while wharf B was for fishermen working on small commercial vessels and traditional vessels. The fishing village mainly was home to artisanal fishermen working on unregistered vessels. Together the sampled participants represented fishermen working on fishing vessels of all types.

To be eligible, participants had to be male, aged 18 years or older, working in fishing full-time (at least six months in the past year), speak Malay, and able to provide written informed consent. At each sampling point, a PWID and a non-PWID were recruited as ‘seeds’, comprising a total of six initial participants. Every participant who completed the study received three coupons to recruit peers from their social network. Coupons were valid for one month from the date of issue and participants had to complete the study at any one of the three sampling locations. No identifying information was collected. Recruitment proceeded in waves, with each wave of participants recruiting the subsequent wave, until sampling reached equilibrium. The criteria for equilibrium were met when the distribution of key variables (HIV and injection drug use status and time spent at sea) changed by less than 2% between recruitment waves.[18]

Participants were remunerated with RM50 (~17USD) for participation and with RM25 (~8USD) for each eligible peer they recruited. This dual-incentive system was designed to recruit large numbers of individuals from otherwise hard-to-reach populations.[13]

Respondents were tested for HIV and HCV, with pre- and post-test counseling provided. Specimens were centrifugally spun down and kept at -20°C until shipped bi-monthly to the University of Malaya laboratory in Kuala Lumpur for analysis.

The questionnaire was self-administered in Malay on a laptop computer, using Questionnaire Development System (QDS) software (Nova Research Company, Maryland, USA). Low- or non-literate participants could self-complete the questionnaire with the use of an audio interface over which questions and response options were read out.

### Measures

#### Socio-Demographic Characteristics

Socio-demographic variables included age, ethnicity, marital status, education and monthly income; responses were dichotomized (see Table 1). Poverty was defined as having a monthly income at or below the poverty line in Malaysia, RM820 (approximately USD273).

**Table 1:** Socio-demographic, occupational and behavioral characteristics and HIV and HCV serological status of Malaysian fishermen (n = 406).

	Sample			Population Estimates	
	Unweighted			RDS Weighted	
	N	%	95% CI	%	95% CI
**Socio-demographic characteristics**					
Age^a^					
25 years and younger	60	14.8	11.3–18.3	18.2	13.1–25.4
Older than 25 years	345	85.0	81.7–88.7	81.8	74.6–86.9
Ethnicity					
Malay	401	98.8	97.7–99.9	98.4	96.8–99.7
Non-Malay	5	1.2	.2–2.3	1.6	.3–3.2
Marital status					
Married	148	36.5	31.8–41.2	30.0	24.0–36.4
Single	258	63.5	58.9–68.3	70.0	63.6–76.0
Education					
Some secondary or lower	279	68.7	64.2–73.3	72.0	66.0–77.0
Completed secondary and higher	127	31.3	26.8–35.8	28.0	23.0–34.0
Monthly income^a^					
At or below poverty line	288	71.6	67.2–76.1	71.8	65.0–77.3
Above poverty line	114	28.4	23.9–32.8	28.2	22.7–35.0
**Occupational characteristics**					
Current vessel type^a^					
Traditional	234	58.1	53.2–62.9	50.3	41.0–59.1
Commercial	169	41.9	37.1–46.8	49.7	40.9–59.0
Occupational role^a^					
Captain	61	15.0	11.7–18.7	12.3	8.7–16.7
Deckhand	341	84.8	81.3–88.4	87.7	83.3–91.3
Time at sea on last fishing trip					
3 days or less	212	52.6	47.7–57.5	50.8	43.6–58.4
4 days or more	191	47.4	42.5–52.3	49.2	41.6–56.4
**Behavioral characteristics**					
Any drug use^a^					
No	216	53.6	48.7–58.5	55.8	47.1–65.2
Yes	187	46.4	41.5–51.3	44.2	34.8–52.9
Sexually active^a^					
No	302	74.9	70.7–79.2	77.1	71.1–81.6
Yes	101	25.1	20.8–29.3	22.9	18.4–28.9
Injection drug use^a^					
No	243	60.3	55.5–65.1	60.7	52.9–69.0
Yes	160	39.7	34.9–44.5	39.3	31.0–47.1
**Serological status**					
HIV					
Negative	358	88.2	85.0–91.3	87.6	83.1–91.6
Positive	48	11.8	8.7–15.0	12.4	8.4–16.9
HCV^a^ ^,^ ^b^					
Negative	202	50.4	45.5–55.3	51.4	42.3–61.1
Positive	199	49.6	44.7–54.5	48.6	38.9–57.7

*Note*: Population estimates from RDS weighted data derived from Respondent Driven Sampling Analysis Tool (RDSAT).

^a^Some missing data due to non-response.

^b^Five samples were found lysed and were not reported.

CI = confidence interval.

#### Occupational Characteristics

We categorized participants according to the vessel type on which they currently worked (commercial vs. traditional), their occupational role (captain vs. deckhand), and the number of days they spent at sea on their previous fishing trip (3 days or less vs. 4 days or more). Working on a commercial vessel was considered to indicate large-scale fisheries and fishing trips of four or more days generally reflected deep-sea fishing.

#### Sexual Behaviors

Participants were asked in separate questions whether they had sex with women and/or men in the past three months. We used these data to compute a dichotomous indicator of sexual activity in the past 3 months. Sexually active participants were subsequently asked if they had traded sex for money or goods, and if they had sex with a sex worker while on a fishing trip, the number of times in the previous three months that they had engaged in vaginal or anal sex with regular and casual female partners, and in anal sex with men. Participants were also asked how many times they had used condoms for vaginal and anal sex with different types of sexual partners in the past three months. For each type of partner, frequency of condom use was subtracted from the frequency of sexual activity to obtain the number of occasions sex occurred without condoms. These data were used to compute the variable ‘inconsistent condom use’ (yes/no).

#### Injection Drug Use

Participants were asked to report whether they had ever used drugs and specified their past and current injection drug use. The Risk Behavior Assessment was used to assess injection-related HIV risk behavior.[19] Respondents who injected drugs were asked the number of times they had engaged in eight unsafe injecting practices in the previous month, with responses for each practice dichotomously recoded to reflect any or no episodes. Participants reporting one or more episodes of unsafe practice were categorized as having engaged in unsafe injecting in the past month (see Table 2). Participants were also asked if they had ever accessed a needle/syringe exchange program and whether they thought they could obtain new and unused needles/syringes when needed.

**Table 2:** Unsafe injecting behavior and needle and syringe exchange access among Malaysian fishermen who inject drugs (n = 160).

	Sample			Population Estimates	
	Unweighted			RDS Weighted	
	N	%	95% CI	%	95% CI
Injected with:					
Used needle	27	16.9	11.0–22.7	14.9	8.9–25.7
Used syringe	31	19.4	13.2–25.6	17.7	9.1–34.2
Drug back-loaded with syringe of someone else	30	18.8	12.6–24.9	15.5	9.1–26.2
Used equipment of someone else	37	23.1	16.5–29.7	25.1	12.7–34.5
Fixed and shared drugs in used equipment	50	31.3	24.0–38.5	29.9	20.1–41.7
Frontloaded syringe from shared equipment	40	25.0	18.2–31.9	22.2	11.7–33.6
Blood of others added to drug during preparation	13	8.1	3.9–12.4	11.5	4.7–20.2
Passing used needle/syringe to others, past month	31	19.4	13.2–25.6	12.1	5.5–20.2
Any unsafe injecting behavior, past month	66	41.3	33.5–49.0	42.5	31.9–55.3

*Note*: Population estimates from RDS weighted data derived from Respondent Driven Sampling Analysis Tool (RDSAT). CI = confidence interval.

#### HIV and HCV Testing

Participants were asked if they had previously tested for HIV and, if so, what the result was of their most recent test. HIV serology was determined using a rapid test (ACON HIV Rapid Test kit, ACON Laboratories, California, USA); reactive results were confirmed with a second rapid test (Intec Products Inc., Xiamen, China). Two reactive results were required for subjects to be classified HIV positive. Two participants had discordant results between the two tests and were classified as HIV negative. HCV serology was determined with ARC Anti-HCV test kits (ThermoFisher Scientific, Massachusetts, USA).

#### Statistical Analyses

We present both unweighted sample means and proportions as well as weighted population estimates from the most commonly used RDS estimator,[20] implemented with the RDS Analysis Tool (RDSAT) version 7.1.38 [21] (S1 File). This estimator uses participants’ recruitment patterns and self-reported personal network size to adjust sample means of study variables based on standardized criteria.[22] To determine personal network size, participants were asked how many fishermen who worked six months in the last year they personally knew and whom they had met at least once in the past six months. RDS estimators enabled adjusting sample means of study variables based on standardized criteria.[22] RDSAT was also used to compute the number of recruitment waves needed to achieve sampling equilibrium, to estimate the average personal network sizes of people who inject drugs (PWID) and non-PWID, and to estimate the proportion of PWID and non-PWID recruiting within their respective groups.[14,20,21,23]

Correlates of HIV and HCV infection and risk behaviors were assessed using both unweighted and RDS weighted data. To generate RDS weighted data, the outcome variables were weighted with RDSAT generated individualized weights prior to analysis.

We used univariate logistic regression analyses to assess socio-demographic, occupational and behavioral correlates of HIV and HCV infection, and socio-demographic and occupational correlates of risk behaviors. To assess independent correlates of HIV and HCV infection, as well as risk behavior, univariately associated variables (*p* < .1) were entered in separate hierarchical multivariate logistic regression models. We entered socio-demographic and occupational characteristics in all models. Risk behavior correlates were added at step two of analyses of HIV and HCV infection. We conducted all statistical analyses with SPSS (version 17; SPSS Inc., Illinois, USA).

## Results

### Recruitment Patterns and Network Characteristics

We recruited and enrolled 406 fishermen over 15 waves. Sample equilibrium was reached after 5 waves. Personal network size ranged from 3 to 150 and averaged 7.4 for fishermen who injected drugs and 8.4 for fishermen who did not inject drugs. Homophily, the tendency to associate with others who were similar to oneself, was moderately low in both groups. Fishermen who injected drugs recruited within their own group 30.5% of the time and fishermen who did not inject drugs recruited within their own group 39.0% of the time.

### Socio-Demographic and Occupational Characteristics

Table 1 shows unweighted sample proportions and RDS-weighted population estimates of socio-demographic characteristics with 95% confidence intervals (CI). Unweighted and RDS-weighted estimates were similar for most characteristics. Age averaged 37.8 years (n = 405; *SD* = 12.2, range 19–78); more than eight in ten fishermen were older than 25 years. Almost all participants were Malay. One-third had completed secondary education. Seventy percent reported an income at or below poverty line. Most participants worked as deckhands, and less than half reported trips at sea of four or more days. Compared to unweighted data, RDS weighted data returned higher proportions of fishermen who were single (63.5% vs. 70.0%, 95% CIs [58.9, 68.3] and [63.6, 76.0] respectively), and worked on commercial vessels (41.9% vs. 49.7%, 95% CIs [37.1, 46.8] and [40.9, 59.0] respectively).

### Sexual Activity and Risk Behaviors

Unweighted and RDS-weighted estimates of sexual behavior were similar (see Table 1). The majority of participating fishermen were not sexually active. Of the sexually active fishermen, one reported sexual intercourse with another man. All others only reported female sexual partners, who were mostly spouses or girlfriends. Fifteen (14.8%) sexually active respondents consistently used condoms. Six men, five with HCV infection and another with HIV infection, reported transactional sex with women. One (HIV negative) participant reporting having sex with female sex workers while on fishing trips.

The one participant who reported having sex with another man reported injection drug use as well as unprotected sex. He tested negative for HIV and positive for HCV. Seven other fishermen reported sex with men in the past but were not currently sexually active with men; three of these men were currently sexually active with women. Six of these individuals also reported injection drug use and tested HCV positive; one was co-infected with HIV.

### Drug Use, Injection and Risk

Unweighted and RDS-weighted estimates of (injection) drug use were highly similar (see Table 1); nearly half (44.2%, 95% CI [34.8, 52.9]) of fishermen were estimated to have used any drugs. The majority (73.8% [63.5, 83.1]) used drugs during fishing trips. More than one-third (39.3% [30.9, 47.1]) of fishermen reported injection drug use. The most commonly injected drugs were heroin, *pil kuda* (an amphetamine type stimulant), and buprenorphine. Most (89.3% [79.9, 95.0]) fishermen who injected drugs reported having accessed a needle and syringe exchange program at least once in their life, and almost all (94.9% [89.9, 98.1]) reported that they thought they could obtain new or unused needles and syringes when needed. However, 42.5% [31.9, 55.3] of fishermen who injected drugs reported unsafe injection practices in the past month (see Table 2).

Multivariate analyses showed that injection drug use was significantly associated with being older than 25 years, being single, working on a commercial vessel, and staying at sea four or more days per trip (see Table 3).

**Table 3:** Socio-demographic, occupational and behavioral correlates of injection drug use among Malaysian fishermen (n = 406).

	Unweighted				RDS Weighted			
	Univariate		Multivariate		Univariate		Multivariate	
	OR	95% CI	AOR	95% CI	OR	95% CI	AOR	95% CI
Being older than 25 years	5.98***	2.64–13.55	9.57***	3.90–23.52	7.00***	3.23–15.15	8.04***	3.49–18.52
Not completed secondary education	1.30	.84–2.01			1.63*	1.03–2.60	1.22	.70–2.12
Being single	2.40***	1.55–3.72	3.00***	1.75–5.10	2.28***	1.42–3.65	3.70***	2.14–6.40
Income on or below poverty line	.32***	.21 –.51	.60^†^	.35–1.04	.36***	.23 –.57	.59^†^	.34–1.02
Fishing on commercial vessel	6.20***	3.99–9.62	3.05***	1.77–5.26	3.85***	2.51–5.89	2.68***	1.59–4.52
Being a deckhand	6.28***	2.78–14.19	3.20*	1.26–8.12	2.61^†^	1.26–5.40	1.87	.80–4.38
Staying at sea 4 days or more^a^	5.34***	3.45–8.26	2.80***	1.63–4.81	3.85***	2.52–5.90	2.04**	1.21–3.46
Nagelkerke R^2^			.412				.341	

*Note*. Respondent Driven Sampling Analysis Tool (RDSAT) weighted injection drug use outcome variable. Univariate and multivariate analyses conducted using logistic regression.

^a^Last trip. OR = odds ratio; AOR = adjusted odds ratio; CI = confidence interval.

^†^ *p* < .1.

* *p* < .05.

** *p* < .01.

*** *p* < .001.

### Testing Positive for HIV or HCV

Estimates of testing positive for HIV or HCV are shown in Table 1. Forty-eight participants (12%) tested positive for HIV and population estimates indicated that HIV prevalence was 12.4% (95% CI [8.4, 16.9]). Estimated HIV prevalence was 24% [13.4, 35.6] among fishermen who injected drugs. Only a third of participants found to be HIV positive knew their status prior to the study.

One hundred and ninety nine fishermen (49%) tested positive for HCV, and population estimates suggest that HCV prevalence was 48.6% (95% CI [38.9, 57.7]). Nearly all (96.9% [93.1, 99.9] fishermen who injected drugs were infected with HCV, but less than a third had previously tested for HCV.

### Correlates of Testing HIV Positive

Factors associated with testing HIV positive were determined using unweighted and RDS weighted data (see Table 4). In univariate analyses, testing HIV positive was significantly associated with age over 25 years, income on or below the poverty line, working on a commercial vessel, staying at sea four or more days per trip and having a history of injection drug use. In addition, for unweighted data a significant association was found between testing HIV positive and being single. No associations were found for education, occupational role and being sexually active. In multivariate logistic regression analysis, only a history of injection drug use remained significantly associated with testing HIV positive. The full models explained 20% (unweighted data) and 23% (RDS weighted data) of the variance in testing HIV positive.

**Table 4:** Socio-demographic, occupational and behavioral correlates of testing HIV positive among Malaysian fishermen (n = 406).

	Unweighted						RDS Weighted					
	Univariate		Multivariate				Univariate		Multivariate			
			Step 1		Step 2				Step 1		Step 2	
	OR	95% CI	AOR	95% CI	AOR	95% CI	OR	95% CI	AOR	95% CI	AOR	95% CI
Being older than 25 years	9.31*	1.26–68.78	9.87*	1.32–74.16	4.69	.6–36.93	8.82*	1.61–48.16	6.80*	1.23–37.67	3.87	.68–22.22
Not completed secondary education	1.61	.79–3.28					1.85	.87–3.96				
Being single	1.84^†^	.92–3.65	2.03*	1–4.11	1.38	.65–2.91	1.66	.80–3.45				
Income on or below poverty line	.56^†^	.30–1.05	.86	.45–1.72	1.11	.55–2.27	.46*	.25 –.86	.65	.33–1.27	.80	.40–1.60
Fishing on commercial vessel	2.87***	1.53–5.38	1.87	.88–3.97	1.2	.53–2.72	1.89*	1.02–3.5	1.23	.60–2.54	.75	.32–1.75
Being a deckhand	2.11	.73–6.11					.84	.35 –.04				
Staying at sea 4 days or more^a^	2.74*	1.44–5.23	1.71	.8–3.64	1.21	.53–2.79	2.60**	1.38–4.91	1.73	.82–3.65	1.61	.68–3.79
Had history of injection drug use	11.62***	5.06–26.67			8.11***	3.23–20.34	8.16***	3.82–17.59			6.22***	2.74–14.13
Being sexually active	1	.5–2.0					.59	.26–1.35				
Nagelkerke R^2^			.124		.234				.094		.198	

*Note*. Respondent Driven Sampling Analysis Tool (RDSAT) weighted testing positive for HIV outcome variable. Univariate and multivariate analyses conducted using logistic regression.

^a^Last trip. OR = odds ratio; AOR = adjusted odds ratio; CI = confidence interval.

^†^ *p* < .1.

* *p* < .05.

** *p* < .01.

*** *p* < .001.

### Correlates of Testing HCV Positive

Analyses of correlates of testing HCV positive using unweighted and RDS weighted data produced similar results (see Table 5). In univariate analyses, testing HCV positive was significantly associated with age over 25 years, not having completed secondary education, being single, income on or below the poverty line, working on a commercial vessel, staying at sea four or more days per trip, a history of injection drug use and being sexually active.

**Table 5:** Socio-demographic, occupational and behavioral correlates of testing HCV positive among Malaysian fishermen (n = 406).

	Unweighted						RDS Weighted					
	Univariate		Multivariate				Univariate		Multivariate			
			Step 1		Step 2				Step 1		Step 2	
	OR	95% CI	AOR	95% CI	AOR	95% CI	OR	95% CI	AOR	95% CI	AOR	95% CI
Being older than 25 years	11.95***	5.72–24.95	14.69***	5.94–36.3	10.05***	2.64–38.36	11.92***	5.57–25.51	14.17***	6.10–32.92	14.35***	3.38–61.04
Not completed secondary education	2.1*	1.35–3.27	1.56	.9–2.72	1.8	.80–3.86	2.1*	1.35–3.28	1.56	.88–2.77	1.44	0.61–3.42
Being single	2.29***	1.51–3.48	3.66***	2.15–6.23	1.93^†^	.95–3.94	2.05***	1.32–3.17	4.15***	2.38–7.23	1.92^†^	.89–4.16
Income on or below poverty line	.35***	.22 - .57	.82	.45–1.46	1.2	.51–2.9	0.35***	0.22–0.56	.63	.35–1.14	.73	.30–1.80
Fishing on commercial vessel	6.44***	4.12–10.07	4.35***	2.42–7.81	2.66*	1.25–5.68	4.02***	2.64–6.11	3.36***	1.94–5.82	2.48*	1.16–5.30
Being a deckhand	1.36	.74–2.5					1.36	.74–2.51				
Staying at sea 4 days or more^a^	5.47***	3.53–8.42	3.49***	2.01–6.05	2.67*	1.32–5.42	5.45***	3.54–8.39	3.19***	1.86–5.49	3.62***	1.72–7.60
Had history of injection drug use	11.95***	5.72–24.95			72.68***	27.43–192.56	149.36***	57.24–389.76			136.89***	44.42–421.80
Being sexually active	2.1*	1.35–3.27			.61	.23–1.6	2.57***	1.57–4.22			.50	0.17–1.49
Nagelkerke R^2^			.462		.731				.454		.771	

*Note*. Respondent Driven Sampling Analysis Tool (RDSAT) weighted testing positive for HCV outcome variable. Univariate and multivariate analyses conducted using logistic regression.

^a^Last trip. OR = odds ratio; AOR = adjusted odds ratio; CI = confidence interval.

^†^ *p* < .1.

* *p* < .05.

** *p* < .01.

*** *p* < .001.

In the full multivariate logistic regression models, testing HCV positive was found to be significantly associated with a history of injection drug use, age over 25 years, and working on a commercial vessel. The association between testing HCV positive and being single was marginally significant, while the association with being sexually active was no longer significant. The full models explained 73% (unweighted data) and 77% (RDS-weighted data) of the variance in testing HCV positive.

## Discussion

To the best of our knowledge, this is the first study using RDS to assess HIV and HCV risks and correlates among fishermen. We acknowledge that there are causes of concern on the performance of RDS in recruiting representative samples and estimating population parameters.[24,25] However, RDS may be less subject to bias than other forms of convenience sampling and we found RDS to be effective in accessing the social networks of a diverse and in some respects difficult to reach population that comprised highly mobile Malaysian fishermen who frequently engaged in illicit drug use. Our use of RDS reflects the realistic necessity to balance precision with feasibility and cost-effectiveness when there are few viable alternatives to study populations for whom no sampling frame is available.[26]

HIV prevalence among Malaysian fishermen in Kuantan was found to be high (12.4%), and similar to the HIV prevalence observed among fishermen in Thailand (15.5%) and Cambodia (16.1%).[4,8,9] Prevalence of HCV was estimated to be even higher, with almost half of fishermen testing HCV positive. Among fishermen with a history of injection drug use, a quarter was HIV positive and almost all were HCV positive. The estimated HIV prevalence among Malaysian fishermen is similar to previous estimates for PWID in Malaysia.[15] HCV prevalence is comparable to estimates for Malaysian PWID in treatment, albeit higher than HCV prevalence in a nationwide sample of Malaysian PWID not in treatment.[27]

Although the high risk of HIV among fishermen in Southeast Asia is generally thought to be related to their sexual behaviors and low frequency of condom use,[8,9,28] our assessment suggests that the main route for HIV transmission among Malaysian fishermen is via injection drug use. A history of injection drug use increased by more than six times the odds of testing positive for HIV. It is of note that most fishermen in this study reported not being sexually active. Possible explanations include that unmarried fishermen have little personal time to seek sexual partners. Furthermore, sex with casual partners is non-normative in Malaysia due to religious and cultural constraints.[29] It is also possible that fishermen have underreported their sexual activity for cultural and other reasons. However, to mitigate such potential bias behavioral data were collected using a self-administered, computerized questionnaire, with an audio interface as required, rather than through face-to-face interviews. Among fishermen who were sexually active, condom use was infrequent with wives or girlfriends. We found little evidence of other practices that could lead to HIV acquisition, such as sex with other men or transactional sex, which were found prevalent among fishermen in other countries.[30,31]

The observed frequency of injection drug use is of note, with 39.3% of fishermen reporting a history of injection drug use. This is considerably higher than found in previous studies among fishermen in Southeast Asia.[32,33] Cross-recruitment between fishermen who did and did not have a history of injection drug use indicates that the social networks of these fishermen were not mutually exclusive. This suggests some level of social acceptability regarding injection drug use in this population of fishermen which may explain the high prevalence of this practice.

Furthermore, unsafe injecting practices were found to be highly prevalent, despite most fishermen who injected drugs reporting accessing NSEPs and indicating they could obtain clean needles/syringes when required. High rates of unsafe injecting are consistent with available data on injection risks among PWID in Southeast Asia, [34] and provide a plausible explanation for the high prevalence of HIV and HCV among these Malaysian fishermen. The study also shows that the occupational context of fishermen constitutes a unique risk environment, with higher frequency of injection drug use reported on commercial vessels and during longer fishing trips.

Injection drug use, being older than 25 years, working on a commercial vessel, and staying at sea for four days or longer per trip were main correlates for HCV infection. While the contribution of injection drug use to HCV acquisition is well established among PWID communities worldwide,[15,35,36] other modes of transmission related to occupational factors and contexts may exist and require elucidation. Working on commercial vessels and engaging in deep-sea fishing require fishermen to spend a longer time at sea as compared to working on traditional vessels and engaging in coastal fishing. These occupational factors and contexts could contribute to an increased risk of HCV acquisition through various routes, including the sharing of razors and toothbrushes.[37,38]

This study has several limitations. Fishermen in Kuantan may not be representative of Malaysian fishermen more generally and findings should be extrapolated with caution. Self-reports of personal network size may be prone to inaccuracies that affected population estimates. However, validity checks were built into the questionnaire to minimize inaccurate responses. As drug use constitutes an illegal practice that could lead to criminalization and imprisonment in Malaysia,[39] injecting drug use may have been underreported. As note above, sexual activity may also be under-reported, in particular sex out of wedlock that is non-normative in Malaysia. Also, sexual activity in the past 3 months may not reflect sexual risk of HIV infection more generally. Assessment of the use of NSEPs was imperfect, as it only captured NSEP access ‘at some point’ and did not encompass patterns of use.

Despite these limitations, this study is the first to provide prevalence estimates of HIV and HCV infection as well as of injection drug use among Malaysian fishermen, in particular in Kuantan. The study findings provide important, novel evidence that Malaysian fishermen are at high risk of HIV as well as HCV acquisition, mostly through unsafe injecting practices. The high prevalence of HIV and HCV infection highlights that the prevention needs of these Malaysian fishermen are not met by existing programs and underscores the importance of strengthening appropriate responses. Increasing the coverage and effectiveness of harm reduction and drug treatment programs for fishermen, especially those working on commercial vessels and engaging in deep-sea fishing, is urgently needed. As many fishermen seemed to be unaware of their HIV and HCV status, scaling-up voluntary counseling and testing services for HIV and HCV in this population is a matter of priority.

## Supporting Information

S1 File Waves Kuantan RDSAT data. (XLSX)
